# Examining Shopping Patterns, Use of Food-Related Resources, and Proposed Solutions to Improve Healthy Food Access Among Food Insecure and Food Secure Eastern North Carolina Residents

**DOI:** 10.3390/ijerph17103361

**Published:** 2020-05-12

**Authors:** Mary Jane Lyonnais, Ann P. Rafferty, Stephanie Jilcott Pitts, Rebecca J. Blanchard, Archana P. Kaur

**Affiliations:** 1 Health Education, Albemarle Regional Health Services, Elizabeth City, NC 27909, USA; 2 Department of Public Health, Brody School of Medicine, East Carolina University, Greenville, NC 27834, USA; raffertya@ecu.edu (A.P.R.); jilcotts@ecu.edu (S.J.P.); kaura19@ecu.edu (A.P.K.); 3 Health Education and Promotion, East Carolina University, Greenville, NC 27834, USA; cookr19@ecu.edu

**Keywords:** food insecurity, food access, rural

## Abstract

In the Southern United States (U.S.), food insecurity rates are higher in rural (20.8%) versus urban communities (15%). Food insecurity can exacerbate diet-related disease. Thus, the purpose of this study was to examine differences in the use of food-related community resources and potential solutions proposed among food insecure versus food secure residents. A community survey (*n* = 370) was conducted in rural eastern North Carolina, with questions pertaining to food security status and food-related resources. The IBM SPSS Statistics software and SAS software were used to examine differences in food-related resources, and qualitative data analysis was used to examine differences in solutions offered between food insecure and food secure participants. Of the 370 respondents, forty-eight-point-six percent were classified as food insecure. Food insecure participants were more likely to report shopping for groceries at a convenience/discount store, less likely to use their own vehicle for transportation, and less likely to purchase food from local producers. Food insecure participants were more likely to suggest solutions related to reducing the cost of healthy food, while food secure participants were more likely to suggest educational or convenience-related interventions.

## 1. Introduction

The U.S. Department of Agriculture (USDA) defines food insecurity as a “lack of consistent access to enough food for an active, healthy life”. [1]. Food insecurity negatively affects health through increases in the prevalence and severity of diet-related disease [2,3,4]. Seligman et al. analyzed a representative sample of National Health and Nutrition Examination Survey participants and found that food insecure participants in the U.S. were 32% more likely to be obese than food secure participants, and severely food insecure individuals were twice as likely to develop type 2 diabetes than their counterparts [5]. U.S. food insecurity rates are higher in rural communities (12.7%) than in urban communities (10.8%) of the U.S. The disparity is greater for the Southern region of the U.S., with non-metropolitan areas having 20.8% food insecure residents compared to metropolitan areas at 15.0% [6]. Therefore, nutrition-related initiatives that address and alleviate food insecurity are needed, particularly in the rural Southern U.S.

There are complex reasons for the urban and rural food insecurity disparity, which could be partially attributed to fewer food-related resources available in rural areas [7,8]. Disparities related to food security in rural environments are impacted by food availability, access, and the relative cost of healthy foods [9]. For example, there are fewer farmers’ markets [10] and less healthy food zoning in rural counties versus urban counties in North Carolina (NC) [11]. Healthy foods may also cost more than less healthy foods in rural areas [12,13]. Due to limited transportation and mobility, food insecure residents may be more likely to shop for food at dollar stores and convenience stores, which have fewer healthy food and beverage options compared to supermarkets and supercenters [14]. Households in the U.S. with annual household incomes of less than $20,000 reported shopping at grocery stores less frequently than those with household incomes higher than $20,000 [15]. Evidence suggests that shopping at small retail stores, as opposed to grocery stores, is correlated with unhealthy purchasing patterns [16,17]. In a study that examined purchasing patterns at small stores in Minneapolis, Minnesota, seventy-one percent of customers purchased at least one or more unhealthy items (such as sugar-sweetened beverages and baked goods), and unhealthy purchases were associated with higher BMIs [18].

Potential solutions to alleviate food insecurity and increase healthy food access among rural populations include farmers’ markets [19,20,21], community supported agriculture [22,23], and community gardens [24]. Learning more about food-related behaviors among food insecure versus food secure individuals is important to inform interventions. Thus, this paper had the following three objectives: (1) to examine differences in shopping patterns among rural residents by food insecurity status; (2) to examine use of food-related community resources by food insecurity status; and (3) to examine the differences in potential solutions to improve healthy food access proposed by food insecure versus food secure residents.

## 2. Materials and Methods

Beaufort County is a rural county in eastern North Carolina with high rates of poverty. The median household income from 2013 to 2017 was $41,101, and the percentage of households receiving Supplemental Nutrition Assistance Program (SNAP) benefits in 2012–2016 was 19.8% [25]. In 2017, sixteen percent of Beaufort County residents were food insecure as defined by the United States Department of Agriculture (USDA), while 20.7% of children in Beaufort County were food insecure [26].

In 2013, a food assessment was conducted in Beaufort County, which ultimately recommended five strategies to improve access to healthy foods, including (1) increasing opportunities for the sale of local products, (2) supporting community and school garden development that engages youth, (3) expanding existing and new educational opportunities such as cooking classes, (4) providing financial support and assistance for Good Agricultural Practices (GAP) certification for farmers, and (5) continuing to facilitate nutrition and healthy eating connections throughout the county. The 2013 assessment resulted in increased efforts in Beaufort County surrounding healthy eating including the implementation of cooking classes at the local food pantry, the enhancement and addition of the farmers’ markets, and several community garden initiatives.

A community survey focused on food access was conducted in October–December 2018 as part of a follow-up county-wide food assessment. The survey questionnaire was developed based on the previous food assessment community survey conducted in 2013 (mentioned above) with input from community partners such as the Healthy Eating and Active Living (HEAL) collaborative. The questionnaire included items about where respondents shopped for food, how far they traveled and the transportation required to purchase groceries, local food purchasing, and food assistance programs and resources. The questionnaire was self-administered and offered in a paper or online format. The survey was conducted using convenience samples of adult residents recruited from across the county. There was an emphasis on recruiting those living in isolated parts of the county and those with barriers to accessing adequate and healthful foods. Survey respondents were recruited at libraries, food pantries, the county departments of health and social services, a health clinic, a Head Start program, community gathering places, and a large discount superstore. In addition, members of the HEAL collaborative had the opportunity to recruit participants in communities where they lived. Recruitment for the online version of the survey was conducted through the HEAL collaborative email, Beaufort County Community College, and other avenues such as Facebook pages. As an incentive to complete the questionnaire, participants were entered into a drawing to win one of the following: $200, $300, and $500 Food Lion gift cards. The study was reviewed and approved by the East Carolina University Institutional Review Board (UMCIRB 18-001877).

Primary shopping venue was assessed with the question “Where do you shop most often for food items?” with response options including: “Convenience store/gas station”, “Dollar store”, “Grocery store”, “Eat at restaurant/go to drive through”, “Farmers’ market/roadside stand/local producer”, and “Other”. Participants were also asked “How do you usually get to the store to shop for food?” with response options including “Ride from friends/family”, “Walk”, “My own personal car/truck”, “Bike”, “County Van Service”, and “Other”. Use of federal and charitable food assistance programs was assessed with the question: “In the past 30 days, have you or anyone in your household used the following to get food?” with response options being yes/no for each of the following: “Supplemental Nutrition Program for Women, Infants and Children (WIC)”, “Supplemental Nutrition Assistance Program (SNAP) or food stamps”, “Food pantry”, “Gleaned foods”, and “Farmers’ market vouchers”. The USDA defines “gleaned foods” as “the act of collecting excess fresh foods from farms, gardens, farmers’ markets, grocers, restaurants, state/county fairs, or any other sources in order to provide it to those in need” [27]. Participants also were asked “Does your household have a vegetable garden, fruit trees or bushes?”, “Do you have family/friends/neighbors that share homegrown food with you and your family?”, and “Do you purchase food directly from local producers (e.g., farmers/growers)?”.

Use of food-related community resources was assessed by asking participants about their engagement in other food-related activities using the following questions: “In the last 5 years, have you participated in cooking classes in your community?” and “In the last 5 years, have you participated in a community garden (by either growing food in one or eating food from one?” Finally, participants were asked if they had attended a farmers’ market for the first time in the last five years.

Food insecurity was measured using a validated 2 item measure developed by Hager et al. [28], which included the following two statements: (1) “Within the past 12 months, we worried whether our food would run out before we got the money to buy more”. (2) “Within the past 12 months, the food we bought just didn’t last and we didn’t have the money to get more.” Response choices were “often true”, “sometimes true”, and “never true”. The food insecurity dichotomous variable was defined as reporting that at least one of the food security statements was often or sometimes true versus neither statement was often or sometimes true.

Demographic questions were asked about age, gender, race, Hispanic ethnicity, and annual household income. The questionnaire is available from the corresponding author upon request.

### 2.1. Quantitative Data Analyses

Univariate and bivariate analyses were conducted using SPSS (IBM Corporation, Armonk, NY, USA), and logistic regressions were generated using SAS (SAS Institutes, Cary, NC, USA). Logistic regressions with individual food venue or combinations of food venues (yes/no) as the dependent variable and food security status as the independent variable were used to examine associations between food security status and food venue type. Food venues included: convenience stores, farmers’ markets, discount stores, grocery stores, and restaurants. Convenience stores were combined with discount stores in some analyses due to a small sample size. In order to examine differences in shopping patterns and use of nutrition-related community resources comparing food secure versus food insecure rural residents, we used chi-squared tests. This study was limited by the convenience sample of Beaufort County residents, which did not represent all residents of Beaufort County or rural eastern North Carolina.

### 2.2. Qualitative Data Analyses

Differences in potential solutions to healthy food access were examined by comparing the responses of food insecure and food secure residents to the open-ended survey question, “What do you think would make it easier for people in your community to buy and eat healthy, local foods?” The responses were analyzed using an iterative, qualitative data analysis approach in which two investigators independently developed codes or themes and operational definitions by using key words from the responses. After a consensus on the codebook with codes and operational definitions was established, the same process was used by each of the two investigators to code the responses. Responses were separated by food insecure versus food secure participants.

## 3. Results

### 3.1. Sample Characteristics

Table 1 shows the sociodemographic characteristics of the sample in total, and by food security status. Of the 370 respondents, one-hundred seventy-eight (48.6%) were classified as food insecure, 188 as food secure, and 4 missing responses. The total sample was about 79% female, 51% white, and 42% black, and 45% of respondents had 3–4 people in the household. Food insecure participants tended to be younger, minority, and had lower household income compared to food secure participants (see Table 1).

### 3.2. Differences in Shopping Patterns and Use of Nutrition-Related Resources, Comparing Food Insecure Versus Food Secure Rural Residents

Table 2 shows differences in shopping patterns and use of nutrition-related resources by food security status. Food insecure participants were more likely to shop at a convenience store/gas station or discount store than food secure participants. Food insecure participants were more likely to report getting a ride from friends or family, walking, biking, or using the county van service to get to the store and were more likely to state that transportation made it hard to get to the store for food compared with those who were food secure. Food insecure participants were also more likely than those who were food secure to report that they, or someone in their household, had used WIC, SNAP, a food pantry, or gleaned foods in the past 30 days. However, among those who were food insecure, fifty-three-point-two percent reported SNAP use and 35.7% food pantry use. Food insecure participants were less likely to have a vegetable garden/fruit trees, less likely to report having a friend or family member share homegrown produce, and less likely to report purchasing food directly from local producers compared with those who were food secure. There were no statistically significant differences by food insecurity status in the proportion who had participated in a community cooking class or community garden or had attended a farmers’ market for the first time in the past five years (see Table 2).

In logistic regression analyses adjusting for zip code of residence, age, race/ethnicity, and income, those who were food insecure had significantly lower odds of primarily shopping at a grocery store compared to those who were food secure (adjusted odds ratio (AOR) = 0.39, 95% CI= 0.16, 0.94, *p* = 0.0347). However, in a similar logistic regression analysis, after adjusting for zip code, age, race/ethnicity, and income, the odds of primarily shopping at a convenience store or discount store for food were not significantly different among those who were food insecure versus food secure (AOR = 1.88, 95% CI = 0.71, 4.96, *p* = 0.2015).

### 3.3. Qualitative Results

Of the total 370 survey participants, two-hundred sixty-one responded to the question about possible solutions to the challenge of increasing access to healthy foods in Beaufort County, “What do you think would make it easier for people to buy and eat healthy, local foods?” There were 273 separate solutions proposed, as some participants mentioned more than one solution. Based on the total possible solutions suggested by respondents, the following 12 themes were found: the addition of a grocery store, farmers’ market, increased education, awareness/advertising, transportation, community interventions, reduce the cost of healthy foods, community gardens, SNAP/Electronic Benefit Transfer (EBT) availability, distance, convenience, and quality of healthy foods in stores.

Approximately half (*n* = 132) of respondents to this question were food insecure. Comparisons of the themes that emerged by food insecurity status highlighted largely different ideas on solutions to increasing access to healthy foods. In particular, six themes emerged that demonstrated contrasts between solutions proposed by those who were food insecure and those who were food secure. The three themes that were most commonly identified by food insecure residents were cost, adding a grocery store to the area, and increasing SNAP/EBT availability. The three themes that were most commonly identified among those who were food secure were educational programming, increasing convenience of healthy foods, and increasing quality of healthy food in stores.

Lastly, there were three themes that were expressed equally between the two groups and three that had varied response rates and no large disparities between food insecure and secure residents. The three that were equally distributed were community gardens, increasing awareness of local healthy options, and community interventions. The remaining three themes were distance, transportation, and farmers’ markets. Table 3 compares the differences in potential solutions to access to healthy foods between food secure and food insecure residents.

## 4. Discussion

Our results indicated that food insecure residents were more likely to use convenience stores and dollar stores for grocery shopping compared with those who were food secure. These findings were in agreement with those of Kaiser et al., who found that food insecure respondents were more likely to shop at a convenience store for food than those who were food secure [29]. This suggested that interventions in convenience and dollar stores were important for improving healthy food intake among food insecure, rural residents. For example, research in seven states indicated that if the Department of Agriculture’s 2016 final rule on SNAP-authorized retailer stocking requirements (which recommends stores that accept SNAP benefits also have a minimum level of stock for several healthy foods) were implemented widely, there would be positive impacts on healthy food options available in SNAP-authorized stores [30]. Research suggests similar efforts are needed in dollar stores, since these stores have no fresh fruit and vegetable options [31], and retailers such as Dollar General are trying to make healthier foods more accessible [32]. Efforts such as these should be evaluated to determine if they will lead to healthier purchases among customers.

Food insecure participants were more likely to use federal food assistance programming such as SNAP and WIC and more likely to use charitable food assistance programming such as food pantries. This highlights the importance of educational programming and policies to ensure that both federal food assistance and charitable food assistance programs provide healthy food and beverage options. Although there are several efforts underway throughout the U.S. to provide healthier foods in food pantries [33,34], still only a small proportion of food insecure residents use the food pantry. Fong et al. suggested that cultural barriers, stigma, and perception of need to justify food pantry use could be reasons why food insecure residents do not use food pantries [35]. More information on attitudes and beliefs about food pantries is needed among food insecure individuals to inform methods to increase reach.

Food insecure participants were less likely to have a vegetable garden/fruit trees, less likely to report having a friend or family member share homegrown produce, and were less likely to report purchasing food directly from local producers. This is important as studies have shown that vegetable gardening is associated with increased vegetable intake among both food insecure and food secure populations and their families in rural areas [36,37]. The reasons for these differences are unknown, but could be related to a lack of financial resources among food insecure participants, a lack of land to grow healthy foods, or a lack of social connections with those in their community who grow healthy food [38].

There was equal use of community nutrition resources among food insecure and food secure participants in terms of attendance at community cooking classes, farmers’ markets, and participation in community gardens. These resources likely improve diet, as well as social connectedness and other positive social outcomes. For example, shopping at farmers’ markets is associated with greater produce intake [17,18]. Armstrong found that not only did community gardens promote healthy behaviors among the participants, but that gardens located in low-income neighborhoods were four times as likely to lead to other neighborhood issues being addressed compared to gardens in higher income areas [39]. Evaluations of cooking classes have determined that such classes can improve self-efficacy for cooking healthier foods, as well as intake of healthier foods [40,41,42]. It is noteworthy that there were no significant differences between food insecure and food secure participants with regards to use of community nutrition resources as this demonstrated that these resources could help reduce health disparities related to food insecurity.

While this study did not assess the impact of discount supermarkets, such food venues are potentially an important feature of the food environment. The county that this study was set in did not have any discount supermarkets. However, the current study’s qualitative data indicated that the addition of a grocery store and lowering the cost of healthy foods were considerations for potential intervention strategies. Lowering the cost of healthy foods such as fruits and vegetables in supermarkets has been shown to be an effective method to increase the purchase of healthy food [43,44,45,46]. This intervention strategy could be used in future studies in rural areas of the U.S. to increase healthy eating habits and reduce food insecurity.

This study was conducted before the outbreak of the novel coronavirus in the U.S.; however, it is likely that the pandemic will affect food security rates in Beaufort County and the rest of northeastern North Carolina. Preliminary data from the University of Arkansas indicated that Southern and mid-Southern regions of the U.S. are experiencing more food insecurity during the pandemic than other regions of the U.S. [47]. Feeding America predicts the economic impact of the coronavirus will be the most severe in low-income households that may already be facing food insecurity and diet-related disease [48]. As unemployment rates continue to rise in North Carolina, it is anticipated that food insecurity rates and associated health effects will as well.

As acknowledged in the methods, this study was limited by the convenience sample of Beaufort County residents, which did not represent all residents of Beaufort County or rural eastern North Carolina. Thus, the statistical results evaluated differences among subgroups of the sample of persons that responded to the survey and not subgroups of the Beaufort County population. A further limitation was the use of self-reported outcome data. The strengths of this study included use of both quantitative and qualitative methods and the use of validated food security questions. Future studies are needed to determine the most effective strategies to reduce food insecurity among rural populations.

## 5. Conclusions

Results from this study suggested that differences among food insecure and food secure populations could affect proposed strategies to improve healthy food access. Therefore, it is critical to involve food insecure participants in all stages of the implementation and evaluation of interventions to improve healthy food access in rural communities.

## Figures and Tables

**Table 1:** Demographic distributions of all participants and by food security status (*N* = 370) *.

Demographic Characteristics	Among All Participants % (*n*)	Within Food Insecurity Status	
		Food Insecure % (*n*)	Food Secure % (*n*)
Age in Years			
19–25	9.6 (35)	11.0 (19)	8.1 (15)
26–35	24.8 (90)	24.9 (43)	24.7 (46)
36–45	14.9 (54)	17.9 (31)	12.4 (23)
46–55	16.3 (59)	22.5 (39)	10.8 (20)
56–65	18.5 (67)	15.6 (27)	21.0(39)
≥66	16.0 (58)	8.1 (14)	23.1 (43)
			
Gender			
Male	21.3 (77)	21.6 (37)	21.0 (39)
Female	78.7 (284)	78.4 (134)	79.0 (147)
			
Race/Ethnicity			
White	51.4 (181)	36.3 (61)	65.6 (118)
Black	42.3 (149)	56.0 (94)	29.4 (53)
Hispanic	4.0 (14)	4.2 (7)	3.9 (7)
Other	2.3 (8)	3.6 (6)	1.1 (2)
			
**Household Income**			
<$10,000	16.5 (60)	28.4 (50)	5.4 (10)
$10,000–24,999	23.6 (86)	33.5 (59)	13.5 (25)
$25,000–39,999	10.2 (37)	11.9 (21)	8.6 (16)
$40,000–54,999	7.4 (27)	6.8 (12)	8.1 (15)
≥$55,000	23.4 (85)	4.5 (8)	41.1 (76)
Prefer not to say	19.0 (69)	14.8 (26)	23.2 (43)
			
Number of People in the Household			
1	11.5 (41)	9.0 (15)	13.9 (26)
2	30.3 (108)	27.1 (45)	32.1 (60)
3–4	44.8 (160)	45.2 (75)	44.9 (84)
≥5	13.4 (48)	18.7 (31)	9.1 (17)
			

Note: *—Numbers do not all add to 370 due to missing responses; %—percent; n—number of participants.

**Table 2:** Differences in shopping patterns and use of nutrition-related resources by food insecurity status (*N* = 370) *. WIC, Supplemental Nutrition Program for Women, Infants and Children; SNAP, Supplemental Nutrition Assistance Program.

Shopping Patterns	Among All Participants % (*n*)	Within Food Insecurity Status		
		Food Insecure % (*n*)	Food Secure % (*n*)	*p*-Value
Place Where Most Often Shops for Food				
Convenience store or gas station	1.9 (7)	3.4 (6)	0.5 (1)	0.001
Discount store	8.4 (31)	13.6 (24)	3.7 (7)	
Grocery store	85.4 (316)	78.0 (138)	93.1 (175)	
Farmers’ market, roadside stand, or from local producer	2.7 (10)	4.0 (7)	1.6 (3)	
Other places	1.1 (4)	1.1 (2)	1.1 (2)	
				
Usual Transportation to Shop for Food				
Ride from friends or family	11.4 (42)	19.9 (35)	3.7 (7)	<0.001
Own personal vehicle	85.6 (314)	75.0 (132)	95.2 (179)	
County van service	0.5 (2)	1.1 (2)	0.0(0)	
Walk	1.9 (7)	2.8 (5)	1.1 (2)	
Bike	0.5 (2)	1.1 (2)	0.0 (0)	
				
Average Travel Time to Food Store				
1–10 min	49.9 (179)	44.4 (76)	55.1 (102)	0.108
11–20 min	31.2 (112)	33.9 (58)	29.2 (54)	
21–30 min	12.0 (43)	12.3 (21)	11.4 (21)	
31–60 min	7.0 (25)	9.4 (16)	4.3 (8)	
				
Transportation makes it difficult to get groceries	13.4(48)	21.5 (37)	6.0 (11)	<0.001
				
Anyone in household used WIC in past 30 days	16.6 (53)	22.3 (33)	11.8 (20)	0.013
				
Anyone in household used SNAP in past 30 days	33.7 (112)	53.2 (84)	15.7 (27)	<0.001
				
Anyone in household used a food pantry in past 30 days	20.2(66)	35.7 (56)	6.0 (10)	<0.001
				
Anyone in household used gleaned foods in past 30 days	3.8 (12)	7.5 (11)	0.6 (1)	0.002
				
Anyone in household used farmers’ market vouchers in past 30 days	1.9 (6)	3.5 (5)	0.6 (1)	0.069
				
Household has vegetable garden, fruit trees, or bushes	19.5 (71)	9.0 (16)	29.7 (55)	<0.001
				
Family, friends, or neighbors share homegrown food with your household	35.1 (129)	24.9 (44)	44.9 (84)	<0.001
				
Purchase food directly from local producers	29.1 (105)	14.8 (26)	42.6 (78)	<0.001
				
Participated in community cooking classes in past 5 years	11.5 (42)	8.6 (15)	13.3 (25)	0.156
				
Participated in community garden in past 5 years	11.3 (41)	12.5 (22)	10.3 (19)	0.516
				
Attended a farmers’ market for the first time in past 5 years	24.4 (88)	23.3 (40)	25.3 (47)	0.657

Note: *—Numbers do not all add to 370 due to missing responses; %—percent; n—number of participants.

**Table 3:** Differences in potential solutions proposed, comparing food insecure and secure residents. EBT, Electronic Benefit Transfer.

Code/Theme	Operational Definition and Illustrative Quote	Food Insecurity Status		
		All Responses (*n*)	Food Secure (*n*)	Food Insecure (*n*)
Grocery Store	Statements that use the words grocery store or store or indicate starting a store in the area that they live, “It would help if we had a grocery store in our community, at this point in time we have to drive at least 20 min to get to the nearest one or go to the nearby dollar store to get what they have and there are no fresh vegetables or fruit and the only meat you can buy is the prepackaged processed kind”	36	12	24
Farmers’ Market/Stand	Statements that use the words farm, farmers’ market, stand or produce stand, suggest increasing the amount of farmers’ markets/produce stands in the area or enhancing them, “Have farmer’s markets in more than one location and on multiple days of the week”	48	27	21
Education	Statements that use the words class, education, preparation or imply teaching someone about either nutrition, healthy lifestyles, or cooking, “They need to understand how to use the fresh food—this can be accomplished with cooking classes/tastings/demos, etc.”	17	12	5
Awareness	Statements that use the words awareness, advertising, publicity, promoting, or suggest getting the word out, “Advertising the local farmers’ markets more so that people are aware of their existence”	27	13	14
Transportation	Statements that use the word transportation, transit, vehicle, or mention getting to a place as a barrier, “Community grocery shopping day with transportation”	13	8	5
Community	Statements that use the word community, co-ops, or mention strengthening community between farmers or between farmers and consumers, “Local sharing programs co-ops etc...”	7	4	3
Cost	Statements that use the words cost, affordable, expensive, cheaper, or prices in regard to making healthy foods less expensive, “Drop prices. All healthy food is expensive”. “It’s cheaper to eat unhealthy”	61	20	41
Gardening	Statements that use the word garden or gardens that can be used by the public or community, “I teach school and feel (Chocowinity Primary School) would be a great place to have a community garden set up to be managed over the summer”	12	6	6
Income Supplements	Statements that use the word SNAP/EBT, food stamps, benefits, WIC, or voucher, “More food stamps” “Vouchers for low-income to get what they need”	11	3	8
Distance	Statements that include the word distance, closer, or shorter travel times to get there, “We have no grocery store or Farmer’s Market with 25 miles of us”	12	5	7
Convenience	Statements that use the words convenient, easy, or strategies that include delivery, “More convenient locations”	15	11	4
Quality	Statements that use the words quality, better, variety, selections, options, or describe produce that indicates it is of good quality and that there are many types to choose from, “Fresh produce that is not wilted or spoiled”	14	12	2
Total		273	133	140

Note: *—Numbers do not all add to 370 due to missing responses; %—percent; n—number of participants.
